# The Impact of Penile Prosthesis Type on Early Postoperative Pain: A Prospective Comparative Study

**DOI:** 10.3390/jcm15145594

**Published:** 2026-07-16

**Authors:** Iyimser Üre

**Affiliations:** Department of Urology, Faculty of Medicine, Eskisehir Osmangazi University, 26040 Eskisehir, Turkey; iyimserure@gmail.com; Tel.: +90-53-0347-3301

**Keywords:** erectile dysfunction, penile prosthesis, malleable penile prosthesis, inflatable penile prosthesis, postoperative pain, patient satisfaction

## Abstract

**Background/Objectives:** Postoperative pain is an important determinant of early recovery and patient experience after penile prosthesis implantation. Although malleable, two-piece inflatable, and three-piece inflatable penile prostheses differ substantially in their mechanical characteristics, data comparing postoperative pain profiles among these devices remain limited. This study aimed to evaluate early postoperative pain perception following penile prosthesis implantation and compare pain outcomes according to prosthesis type. **Methods:** In this prospective observational study, 166 patients who underwent penile prosthesis implantation between January 2020 and December 2025 were evaluated. Patients were divided into three groups according to prosthesis type: malleable penile prosthesis (MPP; *n* = 35), two-piece inflatable penile prosthesis (2IPP; *n* = 72), and three-piece inflatable penile prosthesis (3IPP; *n* = 59). Postoperative pain was assessed using the visual analog scale (VAS) on postoperative day (POD) 1, 7, and 30. Total postoperative non-steroidal anti-inflammatory drug (NSAID) consumption was expressed as ibuprofen-standardized dose (mg). Multivariable linear regression analyses were performed to identify independent predictors of postoperative pain. **Results:** VAS scores on POD1 were comparable among the three groups (*p* = 0.61). However, significant differences emerged during follow-up. On POD7, both the MPP and 2IPP groups demonstrated significantly higher pain scores compared with the 3IPP group (*p* = 0.018 and *p* = 0.041, respectively). On POD30, pain scores remained significantly higher only in the MPP group, whereas no significant difference was observed between the 2IPP and 3IPP groups. Total postoperative NSAID consumption was highest in the MPP group and lowest in the 3IPP group (*p* < 0.001). In multivariable analysis, both MPP implantation (β = 0.95, 95% CI: 0.48–1.42; *p* < 0.001) and 2IPP implantation (β = 0.73, 95% CI: 0.24–1.22; *p* = 0.004) were independently associated with higher POD7 pain scores. For POD30, only MPP implantation remained an independent predictor of increased pain (β = 1.12, 95% CI: 0.58 –1.66; *p* < 0.001). **Conclusions:** Prosthesis type was associated with different postoperative pain trajectories after penile prosthesis implantation. While early postoperative pain appears similar across devices, malleable prostheses are associated with more persistent pain and greater analgesic requirements. Among the evaluated devices, three-piece inflatable prostheses were associated with the most favorable postoperative pain profile. These findings suggest that postoperative patient comfort should be considered alongside functional and technical factors when selecting a penile prosthesis.

## 1. Introduction

Erectile dysfunction (ED) is a common health problem that significantly affects quality of life in men and becomes more prevalent with age [1]. Today, various conservative treatment options—such as oral phosphodiesterase type 5 inhibitors, intracavernosal injection therapies, and vacuum erection devices—are the most commonly preferred treatment modalities [2]. In addition, several regenerative and restorative approaches, including low-intensity shock wave therapy (LiSWT), platelet-rich plasma (PRP), botulinum toxin (BTX), and autologous immune cell-based therapies, have been investigated. However, the long-term efficacy of these approaches remains uncertain, and they are still considered investigational for most patients with ED [3]. For patients who do not respond to or are not suitable candidates for conservative treatment, penile prosthesis implantation remains the definitive surgical treatment for refractory ED. Currently, there are three main types of penile prostheses in clinical use: malleable (semi-rigid) prostheses, two-piece inflatable prostheses, and three-piece inflatable prostheses [2]. These devices differ in their mechanical characteristics, surgical complexity, patient usability, and cost. Thanks to technological advancements and improvements in surgical techniques, penile prosthesis implantation has become a reliable surgical treatment method associated with high patient satisfaction and long-term device success [4].

For many years, success in penile prosthesis surgery was primarily evaluated based on parameters such as the absence of complications—including infection and the need for revision—and the mechanical durability of the prosthesis. However, as patient-centered outcomes have gained increasing importance in recent years, the postoperative recovery process, patient satisfaction, and patient experience have also begun to be recognized as key components of treatment success [5,6]. In this context, postoperative pain is considered one of the primary factors affecting patients’ return to daily activities, early-stage satisfaction, and overall treatment experience [7]. Although pain following penile prosthesis implantation typically subsides over time in most patients, the severity of pain and the recovery process can vary significantly among patients. Additionally, some patients may experience higher-than-expected pain levels or prolonged pain complaints in the early postoperative period, which can negatively impact patient satisfaction and their perception of treatment [8].

In recent years, various strategies have been developed to reduce pain following penile prosthesis implantation. In line with current approaches aimed at reducing opioid use, multimodal analgesia protocols have also become widely adopted in penile prosthesis surgery. It has been reported that these protocols—which include nonsteroidal anti-inflammatory drugs, acetaminophen, gabapentinoids, and various regional nerve blocks—can improve pain control in the early postoperative period and reduce the need for opioids [9,10]. However, most previous studies have focused primarily on optimizing postoperative analgesia rather than evaluating whether prosthesis design itself influences postoperative pain. In particular, there are very few studies in the literature comparing pain profiles among malleable, two-piece inflatable, and three-piece inflatable prostheses. Given the substantial differences in the mechanical characteristics of available prostheses, it is plausible that device type may influence postoperative pain perception [4]. Nevertheless, studies directly comparing the pain profiles of these devices during the early and late postoperative periods are quite limited. This situation makes it difficult to fully understand the potential role of pain in prosthesis selection and the effects of different device types on patient comfort.

Therefore, this prospective observational study aimed to evaluate early postoperative pain following penile prosthesis implantation and to compare pain trajectories among patients receiving malleable, two-piece inflatable, and three-piece inflatable prostheses.

## 2. Materials and Methods

### 2.1. Study Design and Patient Population

This prospective observational study included patients with erectile dysfunction who underwent penile prosthesis implantation at the Department of Urology, Faculty of Medicine, Eskişehir Osmangazi University, between January 2020 and December 2025. The study was approved by the Eskişehir Osmangazi University Ethics Committee for Non-Interventional Clinical Research (Decision No. 138.19) and was conducted in accordance with the principles of the Declaration of Helsinki. Written informed consent was obtained from all patients prior to the study.

All patients who underwent penile prosthesis implantation during the study period and completed postoperative pain assessments were included in the study. Patients who underwent both primary implantation and revision surgery were included in the evaluation. Patients with incomplete clinical data or incomplete postoperative follow-ups were excluded from the study.

A total of 166 patients were included in the study. Patients were divided into three groups based on the type of prosthesis implanted: the malleable prosthesis group (*n* = 35), the two-piece inflatable prosthesis group (*n* = 72), and the three-piece inflatable prosthesis group (*n* = 59).

Demographic and clinical data included age, body mass index (BMI), etiology of erectile dysfunction, diabetes mellitus, hypertension, coronary artery disease, history of pelvic surgery, smoking status, and history of revision surgery. Surgical data included operative time, implant type, reservoir placement, intraoperative findings, and postoperative complications.

All patients were evaluated on postoperative days 1, 7, and 30 as part of the standard postoperative pain assessment protocol. The minimum follow-up period for the study’s primary endpoint was set at 30 days. In addition, patients were monitored for device-related complications and clinical course as part of routine outpatient follow-ups, with a mean clinical follow-up duration of 28.4 ± 14.7 months.

The study protocol was established before patient enrollment and prospectively defined the postoperative pain assessment schedule (postoperative days 1, 7, and 30), analgesic recording procedures, and the primary and secondary study endpoints.

### 2.2. Surgical Technique and Perioperative Management

All surgical procedures were performed by a single surgeon (I.U.) experienced in penile prosthesis surgery using a standardized surgical technique. The penoscrotal approach was preferred in all cases.

In the malleable prosthesis group, Rigicon Rigi10 (Rigicon Inc., Ronkokoma, NY, USA), Coloplast Genesis (Coloplast Corp., Minneapolis, MN, USA), and AMS Spectra (Boston Scientific, Marlborough, MA, USA) prostheses were used. In the two-piece inflatable prosthesis group, all patients were implanted with the AMS Ambicor (Boston Scientific, Marlborough, MA, USA). In the three-piece inflatable prosthesis group, Rigicon Infla10 (Rigicon Inc., Ronkokoma, NY, USA), Coloplast Titan (Coloplast Corp., Minneapolis, MN, USA), and AMS 700 (Boston Scientific, Marlborough, MA, USA) prostheses were used.

Prosthesis type was determined through shared decision-making between the surgeon and the patient. In routine clinical practice, financial considerations represented the primary determinant of prosthesis selection, as penile prostheses are not reimbursed by the Turkish national health insurance system and are therefore purchased directly by patients. Additional factors influencing device selection included the presence of corporal fibrosis (e.g., Peyronie’s disease or a history of priapism), in which narrow-diameter malleable prostheses were often preferred because of challenging corporal dilation. Malleable prostheses were also preferentially selected in some elderly patients and in selected individuals considered to be at increased risk of postoperative infection, based on the surgeon’s clinical judgment.

In patients who received three-piece prostheses, reservoir placement was planned according to patient characteristics. In patients with a history of previous radical pelvic surgery, the reservoir was implanted in an ectopic/submucosal position, while the Retzius space was the standard choice for other patients.

All patients received a single dose of intravenous vancomycin prophylactically during anesthesia induction. In patients with diabetes mellitus, those undergoing revision surgery, or those deemed to be at high risk of infection based on the surgeon’s clinical assessment, prophylactic antibiotic therapy was continued postoperatively with a third-generation cephalosporin for 72 h.

At the end of the operation, a closed vacuum drainage system (MiniVac^®^, Minivac Vacuum Pumps, Mumbai, India) was placed in all patients, and the drains were removed on the first postoperative day.

Early postoperative cycling of inflatable prostheses was not routinely performed. The first device cycling was initiated approximately 4 weeks after surgery, provided that adequate wound healing had been achieved, including resolution of postoperative edema and the absence of clinical signs of infection. At that visit, device inflation and deflation were demonstrated by the surgeon, after which patients were instructed to perform cycling every 2–3 days at home.

### 2.3. Postoperative Pain Assessment and Analgesia Protocol

All patients received treatment with a nonsteroidal anti-inflammatory drug (NSAID) for postoperative pain control. Patients were advised to take the NSAID regularly twice daily for the first 72 h after surgery. After the third day, analgesic treatment was continued according to the patients’ needs.

Patients were provided with a standardized pain diary at discharge and were instructed to record the name, dose, and timing of every NSAID taken during the postoperative period. The completed diaries were collected and reviewed during the routine outpatient visit on postoperative day 30. Diary completeness was assessed during this visit, and any unclear entries were clarified with the patient. Because different NSAID formulations were used, cumulative NSAID consumption was standardized using ibuprofen-comparable doses for analytical purposes. As no universally accepted NSAID equianalgesic conversion table exists, comparable anti-inflammatory dose levels reported in standard clinical pharmacology references were used for standardization [11]. Accordingly, 400 mg of ibuprofen was considered comparable to 25 mg of dexketoprofen, 50 mg of diclofenac, and 250 mg of naproxen. The cumulative postoperative NSAID consumption was then expressed as ibuprofen-standardized doses (mg).

Opioid analgesics were administered only when clinically required on the day of surgery and were not administered or prescribed after postoperative day 1. Acetaminophen was not routinely used. All patients were instructed to take NSAIDs twice daily during the first 72 h according to the standardized postoperative protocol. Thereafter, additional NSAID use was permitted on an as-needed basis according to individual pain severity. Consequently, differences in total NSAID consumption primarily reflected additional analgesic requirements beyond the standardized regimen. No patients with contraindications to NSAID therapy were encountered during the study period.

Postoperative pain levels were assessed using a 10-point visual analog scale (VAS). VAS scores were self-reported by the patients using the same 10-point visual analog scale at all assessment time points. On postoperative day 1, pain scores were recorded with physician assistance before hospital discharge. Assessments on postoperative days 7 and 30 were performed during routine outpatient follow-up visits. The primary endpoint of the study was defined as the VAS scores on postoperative days 1, 7, and 30. Secondary endpoints were defined as total NSAID consumption.

### 2.4. Statistical Analysis

Statistical analyses were performed using Jamovi (Version 2.7.4; The Jamovi Project, Sydney, Australia). The distribution of continuous variables was assessed using the Shapiro–Wilk test. Data showing a normal distribution were presented as mean ± standard deviation, while data not showing a normal distribution were presented as median (minimum–maximum). Categorical variables were expressed as counts and percentages.

To compare continuous variables across the three groups, one-way analysis of variance (ANOVA) was used for data following a normal distribution, and the Kruskal–Wallis test was used for data not following a normal distribution. Pairwise group comparisons were performed using Bonferroni-corrected post-hoc analyses when appropriate. For comparisons of categorical variables, the chi-square test or Fisher’s exact test was used. Multivariate linear regression analysis was performed to identify independent factors associated with postoperative pain. The regression model included age, body mass index, presence of diabetes mellitus, history of revision surgery, history of previous pelvic surgery, operative time, and prosthesis type. VAS scores on the 7th and 30th postoperative days were evaluated as dependent variables in separate models. A *p*-value of <0.05 was considered statistically significant.

## 3. Results

A total of 166 patients were included in the study. Thirty-five patients (21.1%) were in the malleable prosthesis group, 72 (43.4%) in the two-piece inflatable prosthesis group, and 59 (35.5%) in the three-piece inflatable prosthesis group. The demographic and clinical characteristics of the groups are summarized in Table 1.

The mean age in the malleable, two-piece, and three-piece prosthesis groups was 73.2 ± 8.4, 64.8 ± 9.1, and 62.5 ± 8.7 years, respectively (*p* = 0.03). Post-hoc analysis demonstrated that patients in the malleable prosthesis group were significantly older than those in both inflatable prosthesis groups, whereas no significant age difference was observed between the two inflatable prosthesis groups. No statistically significant differences were found between the groups in terms of body mass index, prevalence of diabetes mellitus, hypertension, coronary artery disease, smoking status, history of pelvic surgery, and revision surgery rates (*p* > 0.05 for all comparisons).

Surgical characteristics and postoperative complications are shown in Table 2. The mean operative time differed significantly according to prosthesis type, with the longest operative time observed in the three-piece prosthesis group (73 ± 16 min), followed by the two-piece prosthesis group (58 ± 14 min) and the malleable prosthesis group (42 ± 11 min) (*p* < 0.001). In 21 patients (35.6%) who received three-piece prostheses, the reservoir was placed ectopically or submuscularly, while in 38 patients (64.4%), the standard Retzius placement was preferred. No significant differences were observed between the groups in terms of postoperative hematoma, infection, and corpus perforation rates (*p* > 0.05 for all comparisons).

Postoperative pain outcomes are presented in Table 3. No significant difference was found in VAS scores assessed on postoperative day 1 among the malleable, two-piece inflatable, and three-piece inflatable prosthesis groups (5.9 ± 2.1, 5.3 ± 1.9, and 6.1 ± 2.3, respectively; *p* = 0.61).

In contrast, a significant difference was observed between the groups at the 7th postoperative day assessment (*p* = 0.012). Post-hoc analyses revealed that both the malleable prosthesis group and the two-piece inflatable prosthesis group had higher VAS scores compared to the three-piece inflatable prosthesis group (*p* = 0.018 and *p* = 0.041, respectively). No significant difference was found between the malleable and two-piece inflatable prosthesis groups (*p* = 0.18).

In the evaluation on the 30th postoperative day, although pain scores had decreased significantly in all groups, it was observed that VAS scores in the malleable prosthesis group remained significantly higher than those in the other two groups (*p* = 0.001). Post-hoc analyses revealed that the malleable prosthesis group had higher pain scores compared to both the two-piece and three-piece inflatable prosthesis groups (*p* < 0.001 for both comparisons), while no significant difference was observed between the two-piece and three-piece inflatable prosthesis groups (*p* = 0.42).

A significant difference was observed between the groups in terms of total postoperative NSAID consumption (Table 3). Total analgesic consumption (ibuprofen standardized) was highest in the malleable prosthesis group and lowest in the three-piece inflatable prosthesis group (*p* < 0.001). Post-hoc analyses showed that the malleable prosthesis group, in particular, had a greater need for analgesics compared to the other groups. These differences in analgesic consumption were found to be consistent with the pain scores observed on the 7th and 30th postoperative days.

The results of the multivariate linear regression analysis are presented in Table 4. In the model evaluating pain scores on the 7th postoperative day, operative time, malleable prosthesis implantation, and two-piece inflatable prosthesis implantation were identified as independent predictors of higher VAS scores. Both malleable and two-piece inflatable prosthesis implantation were independently associated with higher POD7 pain scores (β = 0.95; 95% CI 0.48–1.42; *p* < 0.001 and β = 0.73; 95% CI 0.24–1.22; *p* = 0.004, respectively). In addition, operative time was independently associated with pain scores on the 7th postoperative day (β = 0.02; 95% CI 0.01–0.04; *p* = 0.030).

In the model evaluating pain scores on the 30th postoperative day, however, only malleable prosthesis implantation remained an independent predictor (β = 1.12; 95% CI 0.58–1.66; *p* < 0.001). No significant association was found between age, body mass index, diabetes mellitus, history of pelvic surgery, revision surgery, operative time, and two-piece inflatable prosthesis implantation and pain scores at 30 days postoperatively.

Revision surgery and a history of previous pelvic surgery were not identified as independent predictors of postoperative pain scores at any of the time points evaluated.

## 4. Discussion

In this study, the relationship between penile prosthesis type and postoperative pain perception was prospectively evaluated, and it was observed that prosthesis type was associated with different postoperative pain trajectories during follow-up. The main finding of our study is that, although similar pain levels were observed in the early postoperative period, different pain patterns emerged depending on the prosthesis type during the follow-up period. On the 7th postoperative day, higher pain scores were observed in both the malleable and two-piece inflatable prosthesis groups compared to the three-piece inflatable prosthesis group; however, on the 30th postoperative day, only the malleable prosthesis group was found to have significantly higher pain scores. Similarly, analgesic consumption was also found to be higher in the malleable prosthesis group. The fact that malleable prosthesis implantation emerged as an independent predictor of prolonged postoperative pain in multivariate analyses suggests that the observed association is not solely attributable to patient or surgical characteristics. Although patients in the malleable prosthesis group were older, age was not identified as an independent predictor of postoperative pain in multivariate analyses. This suggests that implant characteristics may have contributed more substantially to the observed differences than age alone.

It is noteworthy that postoperative pain scores on day 1 were similar across all prosthesis groups in our study. This finding suggests that pain perception in the early postoperative period is largely determined by surgical trauma, corporal dilation, tissue dissection, and the postoperative inflammatory response, rather than by the type of implant used. It has been previously reported that acute pain following penile prosthesis surgery is largely attributable to surgical manipulation, and that similar pain profiles may emerge within the first 24 h, independent of device characteristics [9,10,12]. Furthermore, the fact that a standard surgical technique was applied to all patients in our study, all operations were performed by the same surgeon, and the postoperative analgesia protocol was similar across all groups enhances the comparability of the pain scores observed in the early period. Therefore, the absence of a significant difference between the groups on the first postoperative day can be considered an expected finding.

In our study, higher pain scores were observed on the 7th postoperative day in both the malleable and two-piece inflatable prosthesis groups compared to the three-piece inflatable prosthesis group. This finding suggests that as the acute effects of surgical trauma subside, the impact of device characteristics on patient comfort begins to become more pronounced. Three-piece inflatable prostheses may be perceived as more comfortable by patients during the early recovery period because they can provide more natural flaccidity and reduce the mechanical load on the corporal tissues. However, while malleable implants maintain constant rigidity, two-piece inflatable implants also have a more limited deflation capacity compared to three-piece systems [2,4]. This situation may have contributed to higher pain perception by affecting the adaptation process of the penile tissues during the first postoperative week. Indeed, the fact that both malleable and two-piece inflatable implants were found to be independently associated with pain scores on the 7th postoperative day in multivariate analyses suggests that the observed difference cannot be explained solely by patient characteristics or surgical variables. Interestingly, although operative time was longest in the three-piece inflatable prosthesis group, these patients experienced lower postoperative pain scores. This finding raises the possibility that implant biomechanics and tissue interaction may play a greater role in postoperative discomfort than surgical duration itself.

One of the most striking findings of our study is that, on the 30th postoperative day, pain scores remained significantly higher only in the malleable prosthesis group. Furthermore, this finding was supported by higher analgesic consumption. The lower pain scores observed in the three-piece inflatable prosthesis group may be related to the ability to deflate these devices during the postoperative period and their imposition of less continuous mechanical load on the corporeal tissues [2,13]. Similarly, although higher pain scores were observed on the 7th postoperative day in the two-piece inflatable prosthesis group, the fact that this difference disappeared by the 30th postoperative day suggests that the interaction between the device and the corporal tissues may become less pronounced as tissue healing progresses. This observation is consistent with prospective data showing that pain completely resolves by the second postoperative week in approximately half of patients who undergo implantation of a three-piece penoscrotal inflatable prosthesis [7].

In contrast, malleable prostheses, due to their permanently rigid structure, do not allow for relaxation of the corporeal tissues and may create ongoing mechanical stress throughout the healing process [14,15]. However, the present study was not designed to directly evaluate biomechanical tissue stress, intracorporal pressure, penile edema, or objective inflammatory markers. Therefore, the proposed mechanisms should be regarded as biologically plausible hypotheses rather than demonstrated explanations for the observed differences in postoperative pain. This situation may be one of the possible explanations for the differences in pain that emerge, particularly following the early postoperative inflammatory phase. Indeed, the finding in multivariate analyses that malleable prosthesis implantation is independently associated with pain scores on the 30th postoperative day indicates that the observed relationship cannot be explained solely by variables such as patient characteristics, revision surgery, or operative time.

The lower pain scores observed in the three-piece inflatable prosthesis group in our study may be related to the advantages these devices offer in terms of patient comfort. Numerous previously published studies have reported that three-piece inflatable prostheses are associated with high rates of patient satisfaction [16]. In the series reported by Bernal et al. and Brinkman et al., the vast majority of patients expressed satisfaction with the device, and the balance between natural flaccidity and erectile function was highlighted as a significant advantage [13,17]. Similarly, Wong et al. reported that implant satisfaction is associated not only with erectile function but also with comfort in daily life and the natural feel of the device [18]. In a recent review, the average patient satisfaction rate was reported to be 86.2% for inflatable prostheses and 75.1% for malleable prostheses [6]. Although satisfaction was not directly assessed in our study, it is possible that the lower pain scores observed in the three-piece prosthesis group may be an early reflection of these patient comfort advantages. Considering that postoperative pain is one of the key determinants of the patient experience, it can be argued that the lower pain scores observed in the three-piece prosthesis group may represent one contributing factor to long-term satisfaction.

Revision procedures were intentionally included because they constitute a clinically relevant subgroup of patients undergoing penile prosthesis implantation in routine practice. To minimize potential confounding, revision surgery was included as a covariate in the multivariable regression analysis. Furthermore, it is noteworthy that a history of revision surgery and previous pelvic surgery was not found to be associated with postoperative pain. Although higher pain might be expected, particularly in these cases requiring more complex surgical dissection and reoperative surgery [19], no such association was demonstrated in multivariate analyses. This finding suggests that implant characteristics may contribute more substantially to postoperative pain than surgical complexity in this cohort.

The effect of diabetes and glycemic control on postoperative pain has been investigated previously. Reinstatler et al. reported that unplanned visits due to postoperative pain were more frequent in patients with HbA1c levels above 8%. In contrast, in our study, HbA1c level was not identified as an independent predictor of prolonged pain. This finding may be attributed to the fact that pain assessment was based directly on VAS scores rather than the number of unplanned visits, as well as differences between patient populations [20].

This study has several strengths. First, to the best of our knowledge, this is one of the first studies to prospectively compare the effects of different types of penile prostheses on postoperative pain. The fact that all surgeries were performed by the same surgeon, that a standardized surgical technique was used, and that the same postoperative pain assessment protocol was applied to all patients enhanced the comparability between the study groups. Furthermore, the fact that pain assessment was not limited to the early postoperative period but also included the 7th and 30th postoperative days allowed for an evaluation of changes in the pain course over time.

However, the study also has some limitations. First, the study was single-center and did not employ a randomized design. In our clinical practice, prosthesis selection was primarily influenced by patients’ financial resources. In addition, corporal fibrosis, advanced age, and the surgeon’s assessment of infection risk also influenced device selection. Although multivariable regression was performed to adjust for measured confounders, residual confounding and selection bias cannot be completely excluded. Therefore, the observed associations should not be interpreted as evidence of a causal relationship. Second, patient satisfaction, quality of life, and functional outcomes were not assessed. Consequently, the relationship between postoperative pain and long-term patient satisfaction could not be directly established. Third, since pain assessment in this study was limited to the 30th day, the development of chronic pain could not be evaluated. Finally, the VAS score used in pain assessment is a subjective measurement method and may have been influenced by individual differences in pain perception. Therefore, the observed associations should be interpreted within the context of a prospective observational study and should not be considered evidence of a causal relationship. Future multicenter randomized studies with longer follow-up periods are needed to validate our findings.

## 5. Conclusions

In conclusion, prosthesis type was independently associated with postoperative pain scores in this prospective observational cohort. Although similar pain levels were observed in the early postoperative period in our study, different pain patterns emerged over the follow-up period depending on the prosthesis type. Malleable prostheses were associated with higher pain scores and greater analgesic consumption on the 7th and 30th postoperative days, whereas the pain difference observed with two-piece inflatable prostheses resolved over time. In contrast, three-piece inflatable prostheses were associated with lower pain scores. These findings suggest that not only technical and functional characteristics, but also postoperative patient comfort may be considered when selecting a penile prosthesis. Prospective studies with longer follow-up periods will contribute to a better understanding of the relationship between prosthesis type and long-term pain and patient satisfaction.

## Figures and Tables

**Table 1:** Baseline demographic and clinical characteristics of the study population.

Variable	Malleable Prosthesis (*n* = 35)	Two-Piece Inflatable Prosthesis (*n* = 72)	Three-Piece Inflatable Prosthesis (*n* = 59)	*p*-Value
Age (years)	73.2 ± 8.4	64.8 ± 9.1	62.5 ± 8.7	**0.030**
BMI (kg/m^2^)	29.8 ± 4.1	30.2 ± 4.3	29.5 ± 3.8	0.670
Diabetes mellitus, *n* (%)	15 (42.9)	27 (37.5)	20 (33.9)	0.680
Hypertension, *n* (%)	18 (51.4)	35 (48.6)	27 (45.8)	0.840
Coronary artery disease, *n* (%)	7 (20.0)	12 (16.7)	8 (13.6)	0.710
Current smoker, *n* (%)	11 (31.4)	20 (27.8)	15 (25.4)	0.810
Previous pelvic surgery, *n* (%)	13 (37.1)	27 (37.5)	21 (35.6)	0.980
Revision surgery, *n* (%)	3 (8.6)	12 (16.7)	5 (8.5)	0.240
Vasculogenic ED, *n* (%)	18 (51.4)	35 (48.6)	30 (50.8)	0.960
Diabetic ED, *n* (%)	9 (25.7)	17 (23.6)	13 (22.0)	0.910
Post-radical pelvic surgery ED, *n* (%)	6 (17.1)	15 (20.8)	11 (18.6)	0.870
Peyronie’s disease, *n* (%)	2 (5.7)	5 (6.9)	5 (8.5)	0.840

Bold values indicate statistically significant differences (*p* < 0.05).

**Table 2:** Perioperative characteristics and postoperative complications.

Variable	Malleable Prosthesis (*n* = 35)	Two-Piece Inflatable Prosthesis (*n* = 72)	Three-Piece Inflatable Prosthesis (*n* = 59)	*p*-Value
Operative time (min)	42 ± 11	58 ± 14	73 ± 16	**<0.001**
Length of hospital stay (days)	1.2 ± 0.4	1.3 ± 0.5	1.4 ± 0.6	0.210
Revision surgery, *n* (%)	3 (8.6)	12 (16.7)	5 (8.5)	0.240
Reservoir placement (Retzius), *n* (%)	N/A	N/A	38 (64.4)	N/A
Reservoir placement (Ectopic/Submuscular), *n* (%)	N/A	N/A	21 (35.6)	N/A
Corporal perforation, *n* (%)	1 (2.9)	1 (1.4)	1 (1.7)	0.880
Postoperative hematoma, *n* (%)	2 (5.7)	3 (4.2)	3 (5.1)	0.940
Early prosthesis infection, *n* (%)	1 (2.9)	0 (0)	1 (1.7)	0.770
Device explantation during follow-up, *n* (%)	1 (2.9)	0 (0)	1 (1.7)	0.770

Bold values indicate statistically significant differences (*p* < 0.05). N/A: Not applicable. Reservoir placement was not applicable to malleable and two-piece inflatable prostheses.

**Table 3:** Postoperative Pain Scores (VAS) and analgesic consumption.

Variable	Malleable Prosthesis (*n* = 35)	Two-Piece Inflatable Prosthesis (*n* = 72)	Three-Piece Inflatable Prosthesis (*n* = 59)	*p*-Value
POD1	5.9 ± 2.1	5.3 ± 1.9	6.1 ± 2.3	0.610
POD7	4.8 ± 1.8	4.4 ± 1.9	3.1 ± 1.5	**0.012**
POD30	2.5 ± 1.0	1.4 ± 0.8	1.2 ± 0.7	**0.001**
Total NSAID consumption (mg)	7850 ± 2150	6980 ± 1940	5420 ± 1730	**<0.001**

Values represent cumulative ibuprofen-standardized NSAID doses. Bold values indicate statistically significant differences (*p* < 0.05).

**Table 4:** Multivariable linear regression analysis for predictors of postoperative pain scores.

Variable	POD7 β Coefficient (95% CI)	*p*-Value	POD30 β Coefficient (95% CI)	*p*-Value
Age	−0.01 (−0.03 to 0.01)	0.280	−0.01 (−0.03 to 0.01)	0.310
BMI	0.04 (−0.01 to 0.09)	0.120	0.03 (−0.02 to 0.08)	0.220
Diabetes mellitus	0.18 (−0.15 to 0.51)	0.290	0.12 (−0.21 to 0.45)	0.470
Previous pelvic surgery	0.14 (−0.22 to 0.50)	0.440	0.09 (−0.28 to 0.46)	0.620
Revision surgery	0.19 (−0.26 to 0.64)	0.410	0.15 (−0.27 to 0.57)	0.480
Operative time	0.02 (0.01 to 0.04)	0.030	0.01 (−0.01 to 0.03)	0.180
Two-piece inflatable prosthesis	0.73 (0.24 to 1.22)	0.004	0.18 (−0.12 to 0.48)	0.240
Malleable prosthesis	0.95 (0.48 to 1.42)	<0.001	1.12 (0.58 to 1.66)	**<0.001**

Reference category: Three-piece inflatable prosthesis. Abbreviations: BMI, body mass index; CI, confidence interval; POD, postoperative day. Bold values indicate statistically significant differences (*p* < 0.05).

## Data Availability

The data presented in this study are available on request from the corresponding author.

## Abbreviations

The following abbreviations are used in this manuscript:

ANOVA	Analysis of Variance
BMI	Body Mass Index
CAD	Coronary Artery Disease
CI	Confidence Interval
DM	Diabetes Mellitus
ED	Erectile Dysfunction
HT	Hypertension
IPP	Inflatable Penile Prosthesis
LiSWT	Low-Intensity Shock Wave Therapy
MPP	Malleable Penile Prosthesis
NSAID	Non-Steroidal Anti-Inflammatory Drug
POD	Postoperative Day
PRP	Platelet-Rich Plasma
VAS	Visual Analog Scale
